# Novel Imaging Techniques Prior to Surgical Management of Invasive Bladder Cancer: A Systematic Review

**DOI:** 10.1007/s11934-026-01370-z

**Published:** 2026-10-22

**Authors:** Mahmoud Hijazi, Jacob Hanna, Jennifer Sykes, David Golombos

**Affiliations:** 1 Rutgers Robert Wood Johnson Medical School, New Brunswick, New Jersey USA; 2 Section of Urologic Oncology, Rutgers Cancer Institute, 195 Little Albany Street, New Brunswick, NJ 08903 USA

**Keywords:** Urinary bladder neoplasms, Magnetic resonance imaging, Positron–emission tomography, Cystectomy, Neoplasm staging

## Abstract

**Purpose of Review:**

To assess the diagnostic accuracy of novel imaging modalities in working up invasive bladder cancer prior to surgical management and their potential role in guiding treatment decisions.

**Recent Findings:**

Multiple new imaging modalities, including VI-RADS and Biparametric MRI have been developed to better guide management of invasive bladder cancer. There lacks a comprehensive review of these modalities and their diagnostic performance.

**Summary:**

We performed a systematic review following PRISMA guidelines. PubMed, Embase, and Scopus were searched through 2025. Studies reporting sensitivity and specificity of novel imaging for muscle invasion detection or nodal staging were included. Quality assessment used the QUADAS 2 tool. Thirty three studies encompassing 4,689 patients were included. Twenty three studies evaluated the Vesical Imaging Reporting and Data System for muscle invasion detection, achieving median sensitivity of 94% and specificity of 82% at a threshold of three or greater and 84% and 92%, respectively, at four or greater. Nine studies assessed positron emission tomography for nodal staging, with fluorodeoxyglucose positron emission tomography/computed tomography demonstrating superior sensitivity (23 to 81%) compared to conventional computed tomography (13 to 47%). Fibroblast activation protein inhibitor tracers showed the highest sensitivity (82 to 100%) with specificity of 82 to 90%. One radiomics study achieved 73% sensitivity and 84% specificity for lymph node detection. Novel imaging modalities demonstrate promising diagnostic accuracy for bladder cancer staging. The Vesical Imaging Reporting and Data System provides reliable muscle invasion assessment with threshold dependent performance. Positron emission tomography with fibroblast activation protein inhibitor tracers substantially improves nodal staging over conventional computed tomography. Integration of these modalities may optimize patient management, including use or omission of neoadjuvant therapy and consideration for organ sparing approaches. Prospective multicenter validation is needed.

## Introduction

Bladder cancer is the second most common genitourinary cancer and sixth most common cancer in the United States, with an estimated 84,530 new cases and 17,870 deaths projected in 2026 [1]. Radical cystectomy with bilateral pelvic lymph node dissection remains the standard of care for muscle–invasive bladder cancer (MIBC) and select high–risk non–muscle–invasive disease refractory to intravesical therapy [2]. However, the morbidity associated with radical cystectomy is substantial, with complication rates exceeding 60% in contemporary series and significant impacts on quality of life [3].

Treatment planning depends on accurate preoperative staging, which currently relies on transurethral resection of bladder tumors (TURBT), upper tract imaging, bimanual examination under anesthesia, and cross–sectional body imaging to determine local stage and evaluate for metastatic disease [2]. However, conventional imaging has well–recognized limitations. CT and MRI have limited sensitivity for detecting microscopic muscle invasion, and CT–based lymph node assessment relies on size criteria alone, resulting in sensitivity as low as 13% to 47% for detecting nodal metastases [4]. These diagnostic shortcomings can lead to clinical under staging or over staging, both of which carry significant implications for disease management.

In appropriately selected patients, organ–sparing approaches offer a viable alternative to radical cystectomy. Trimodal therapy, consisting of maximal TURBT, radiation, and concurrent chemotherapy, has demonstrated comparable 5–year overall survival and cancer–specific survival to radical cystectomy in selected populations [5, 6]. Partial cystectomy may also be considered in patients with solitary tumors in favorable locations, those unfit for radical surgery, or those who decline urinary diversion [7]. Additionally, organ–sparing cystectomy represents an emerging option for select women with invasive disease. The success of these strategies hinges on the ability to accurately identify patients with organ–confined disease, making precise preoperative imaging critical to clinical decision–making.

Several novel imaging modalities have emerged to address these diagnostic gaps. The Vesical Imaging–Reporting and Data System (VI–RADS), introduced in 2018, provides a standardized scoring system for MRI–based assessment of muscle invasion [8]. Machine learning and radiomics approaches applied to standard CT imaging represent a scalable alternative for institutions without PET capabilities. Despite the growing number of studies evaluating these modalities, no comprehensive synthesis of their diagnostic performance has been performed.

Utilizing a systematic review, we aimed to assess the diagnostic accuracy of novel imaging modalities in working up invasive bladder cancer prior to surgical management.

## Materials and Methods

### Study Design

This systematic review was conducted in accordance with the Preferred Reporting Items for Systematic Reviews and Meta–analyses (PRISMA) 2020 guidelines [9].

### Literature Search

A comprehensive search of PubMed/MEDLINE, Cochrane Library, EMBASE, and Scopus was performed in 2025 using Covidence, a web–based systematic review management platform. Controlled Medical Subject Heading (MeSH) terms were combined with free–text keywords to optimize search sensitivity. The following strategy was employed:

*“Urinary Bladder Neoplasms” OR “bladder cancer” OR “urothelial carcinoma” AND “Cystectomy” OR “radical cystectomy” AND “Imaging” OR “Preoperative Imaging” OR “Staging Imaging” OR “multiparametric MRI” OR “mpMRI” OR “diffusion–weighted imaging” OR “DWI” OR “dynamic contrast enhanced MRI” OR “radiomics” OR “PSMA–PET” OR “PET–CT” OR “surgical planning.”*

### Study Selection

Studies were included if they assessed the diagnostic accuracy of novel imaging modalities for the determination of muscle invasion and/or staging of bladder cancer, reporting sensitivity and specificity as primary outcomes. Studies evaluating standard imaging alone were excluded unless a novel modality was used as a comparator. Non–English studies, case reports, conference abstracts without full–text data, and editorials were excluded. No date restrictions were applied.

### Article Review Process

Three independent reviewers evaluated study abstracts. For an abstract to advance to full–text review, agreement between at least two of the three reviewers was required. Full–text articles were then assessed by two independent reviewers, and discrepancies were resolved by consensus. Quality assessment was performed using the Quality Assessment of Diagnostic Accuracy Studies–2 (QUADAS–2) tool, which evaluates risk of bias across four domains (patient selection, index test, reference standard, and flow and timing) and applicability concerns across three domains.

### Data Extraction

Data extraction was performed independently by each reviewer using a standardized template. Variables collected included first author, year of publication, country of origin, study design, sample size, imaging modality, patient population, and reference standard. Primary end points were sensitivity and specificity. Secondary end points included positive predictive value, negative predictive value, diagnostic accuracy, and area under the receiver operating characteristic curve.

## Results

**Fig. 1:**
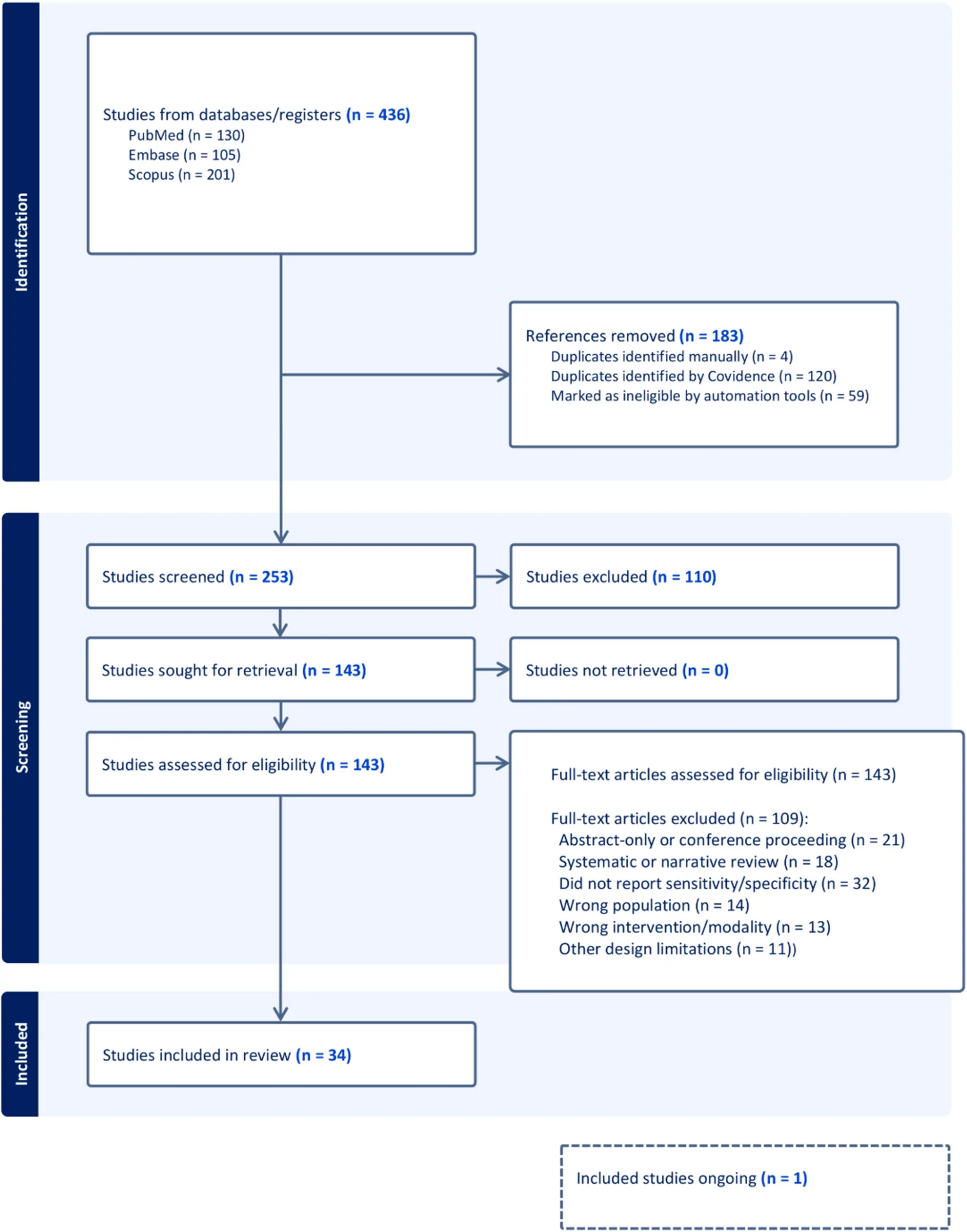
Preferred Reporting Items for Systematic Reviews and Meta–Analyses (PRISMA) flow diagram. A total of 436 records were identified, with 33 studies ultimately included in the review

A total of 436 records were identified through database searches across three major databases (PubMed, *n* = 130; Embase, *n* = 105; Scopus, *n* = 201). Following duplicate removal (*n* = 183), 253 records underwent title and abstract screening. After initial screening excluded 110 studies, 143 full–text articles were assessed for eligibility. Of these, 110 were excluded for the following reasons: abstract–only or conference proceeding (*n* = 21), systematic or narrative review (*n* = 18), did not report sensitivity/specificity (*n* = 33), wrong population (*n* = 14), wrong intervention/modality (*n* = 13), and other design limitations (*n* = 11). Ultimately, 33 studies met all inclusion criteria and were included in this review (Fig. 1).

The 33 included studies encompassed 4,689 patients across diverse clinical settings (Table 1). Vesical Imaging–Reporting and Data System (VI–RADS)–based MRI studies comprised the majority of evidence (*n* = 23, 69.7%), while PET–based imaging approaches accounted for 9 studies (27.3%). One study evaluated CT–based radiomics for lymph node assessment.

**Table 1:** Basic characteristics of included studies

Study	Study Design	Country	Intervention	Population	Reference Standard
VI–RADS Studies					
Ahn et al. [10]	Retrospective	South Korea	VI–RADS	Bladder cancer (mixed)	TURBT/cystectomy
Arita et al. [11]	Retrospective	USA/Japan/Netherlands	VI–RADS (bp vs. mp)	Variant urothelial carcinoma	TURBT/cystectomy
Arita et al. [12]	Retrospective	Japan	VI–RADS	Pure vs. variant UC	TURBT/cystectomy
Aslan et al. [13]	Retrospective	Turkey	VI–RADS	Pure vs. variant UC	TURBT/cystectomy
Bricio et al. [14]	Retrospective	Brazil	VI–RADS (mp vs. bp)	Bladder cancer (confirmed)	TURBT/cystectomy
Del Giudice et al. [15]	Retrospective	Italy	VI–RADS	MIBC	Cystectomy
Dogan et al. [16]	Prospective	Turkey	VI–RADS + shape	Bladder cancer (suspected)	TURBT/cystectomy
Elshewy et al. [17]	Prospective	Egypt	VI–RADS	Bladder mass	TURBT/cystectomy
Gong et al. [18]	Retrospective	China	VI–RADS + morphology	Urothelial carcinoma	TURBT/cystectomy
Gupta et al. [19]	Prospective	India	VI–RADS	Bladder SOL	TURBT/cystectomy
Gungor et al. [20]	Prospective	Turkey	VI–RADS	Bladder cancer (suspected)	TURBT/cystectomy
Huele et al. [21]	Prospective	Netherlands	mpMRI	Invasive bladder cancer	Cystectomy
Islam et al. [22]	Prospective	India	VI–RADS	Treatment–naïve BC	TURBT/cystectomy
Jai–ua et al. [23]	Prospective	Thailand	VI–RADS	Bladder mass	TURBT
Kazan et al. [24]	Retrospective	Turkey	VI–RADS	Bladder cancer (primary)	TURBT/cystectomy
Kim et al. [25]	Retrospective	South Korea	VI–RADS	Pre–cystectomy candidates	Cystectomy
Kong et al. [26]	Prospective	China	VI–RADS + APTw	Bladder cancer (confirmed)	TURBT/cystectomy
Liu et al. [27]	Retrospective	China	VI–RADS	Bladder cancer	TURBT/cystectomy
Metwally et al. [28]	Prospective	Egypt	VI–RADS	Bladder cancer	TURBT/re–TURBT
Özdemir et al. [29]	Retrospective	Turkey	VI–RADS	Bladder cancer (primary)	TURBT/cystectomy
Piramide et al. [30]	Prospective	Italy	VI–RADS (mp vs. bp)	Bladder cancer (suspected)	TURBT/cystectomy
Ueno et al. [31]	Retrospective	Japan	VI–RADS	Pre–TURBT candidates	TURBT/cystectomy
van Koeverden et al. [32]	RCT (ongoing)	Netherlands	VI–RADS + biopsy	Bladder cancer (suspected)	Cystectomy/biopsy
Al–Zubaidi et al. [33]	Retrospective	Australia	18 F–FDG PET vs. CT	Invasive BC (≥ T1)	PLND/biopsy
PET/CT Studies					
Bertolaso et al. [34]	Retrospective	France	18 F–FDG PET vs. CT	MIBC	PLND
Einerhand et al. [35]	Retrospective	Netherlands	FDG PET/CT vs. CT	MIBC/high–risk T1	PLND
Longoni et al. [36]	Retrospective	Italy	18 F–FDG PET/CT	MIBC/BCG–unresponsive NMIBC	PLND
Moussa et al. [37]	Retrospective	Lebanon	18 F–FDG PET vs. CT	MIBC/high–risk NMIBC	PLND
Ng et al. [38]	Prospective pilot	USA	18 F–fluciclovine PET	MIBC (T2–T4)	Cystectomy
Pihl et al. [39]	Retrospective	Sweden	18 F–FDG PET/CT	BC (pre–cystectomy)	PLND
Unterrainer et al. [40]	Retrospective	Germany	68Ga/18F–FAPI PET	MIBC	PLND
van Ginkel et al. [41]	Retrospective	Netherlands	18 F–FDG PET/CT	High–risk NMIBC	Composite
Gresser et al. [42]	Retrospective	Germany	CT radiomics	UC/squamous cell CA	PLND

Abbreviations: *APTw* amide proton transfer weighted, *BC* bladder cancer, *BCG* Bacillus Calmette–Guérin, *bp* biparametric, *CT* computed tomography, *FAPI* fibroblast activation protein inhibitor, *FDG* fluorodeoxyglucose, *MIBC* muscle–invasive bladder cancer, *mp* multiparametric, *NMIBC* non–muscle–invasive bladder cancer, *PET* positron emission tomography, *PLND* pelvic lymph node dissection, *RCT* randomized controlled trial, *SOL* space–occupying lesion, *TURBT* transurethral resection of bladder tumor, *UC* urothelial carcinoma, *VI–RADS* Vesical Imaging–Reporting and Data System.

Study designs were predominantly retrospective cohort studies, with several prospective investigations including one ongoing randomized controlled trial examining VI–RADS–guided diagnostic pathways. Reference standards varied systematically by imaging approach, with VI–RADS studies primarily employing TURBT or cystectomy specimens for pathological correlation, while PET/CT studies used pelvic lymph node dissection (PLND) pathology as the reference standard.

### VI–RADS Performance

Twenty–three studies evaluated 3,041 patients for MIBC through the VI–RADS MRI scoring system [10–32]. VI–RADS is a 5–point Likert scoring system applied to multiparametric MRI that integrates T2–weighted, diffusion–weighted, and dynamic contrast–enhanced sequences to estimate the probability of muscle invasion, with higher scores indicating greater likelihood of MIBC [8**]**. Performance of the VI–RADS scoring system varied by the cutoff score used to identify MIBC. A score of three or greater achieved a median sensitivity of 94% (range 76–100%) and specificity of 82% (range 66–95%). Using a higher threshold of four or greater yielded a median sensitivity of 84% (range 65–100%) and specificity of 92% (range 23–100%) (Table 2).

**Table 2:** Diagnostic accuracy of novel imaging approaches for muscle invasion detection

Study	*n*	Imaging Approach	Threshold	Sens (%)	Spec (%)	PPV (%)	NPV (%)	Acc (%)	AUC	Primary Outcome	Scanner
VI–RADS Studies											
Ahn et al. [10]	82	VI–RADS	≥ 3	79	97	NR	NR	93	0.94	MI detection	3T
Arita et al. [11]	118	VI–RADS (mp/bp)	≥ 4	77–80/65–67	88–92/88–90	NR	NR	82–85/76–77	0.90–0.92/0.87–0.88	MIBC detection	1.5T
Arita et al. [12]	122	VI–RADS VUC vs. PUC	≥ 4	76–79/85–85	89–93/93–93	NR	NR	NR	0.92–0.89/0.94–0.93	PUC vs. VUC	1.5T
Aslan et al. [13]	173	VI–RADS VUC vs. PUC	≥ 4	94–94/87–82	100/95–94	NR	NR	96–96/91–88	0.84–0.78/0.97–0.97	MIBC detection	1.5T
Bricio et al. [14]	44	VI–RADS (mp/bp)	≥ 4	NR	NR	NR	NR	NR	0.89–0.92/0.88–0.92	MIBC detection	1.5T
Del Giudice et al. [15]	149	VI–RADS	≥ 5	90	98	95	96	96	0.94	Extravesical	3T
Dogan et al. [16]	119	VI–RADS + shape	≥ 3/≥4	100/81	66/95	51/86	100/77	75/92	0.75/0.92	MIBC detection	1.5T
Elshewy et al. [17]	80	VI–RADS	> 3	90	83	83	90	NR	0.90	MIBC detection	3T
Gong et al. [18]	198	VI–RADS + morphometry	NR	NR	NR	NR	NR	NR	0.91/0.80–0.86	MIBC detection	3T
Gupta et al. [19]	76	VI–RADS	≥ 3	94	88	87	95	91	NR	MIBC detection	NR
Gungor et al. [20]	63	VI–RADS	> 3	100	23	62	100	66	NR	MIBC detection	3T
Huele et al. [21]	37	mpMRI	cT-stage	88	58	81	70	78	NR	MIBC detection	3T
Islam et al. [22]	60	VI–RADS	≥ 3/≥4	98/85	74/84	89/92	93/73	90/85	NR	MIBC detection	3T
Jai–ua et al.[23]	35	VI–RADS	≥ 3/≥4	100/92	82/87	62/67	100/98	86/88	0.93	MIBC detection	1.5T
Kazan et al. [24]	96	VI–RADS	≥ 3/≥4	85/80	80/88	53/64	95/94	81/87	0.83/0.84	MIBC detection	1.5T
Kim et al. [25]	159	VI–RADS	≥ 4/≥5	84/78	93/99	84/96	93/95	90/95	0.88/0.89	MI/Extravesical	3T
Kong et al. [26]	106	VI–RADS + APTw/ADC	NR	84–91	84–85	NR	NR	84–93	0.84–0.93	MIBC detection	3T
Liu et al. [27]	126	VI–RADS	≥ 4	94	92	89	96	NR	0.97	MIBC detection	3T
Metwally et al. [28]	331	VI–RADS	> 3	84	92	93	84	88	NR	MIBC detection	1.5T
Özdemir et al. [29]	283	VI–RADS	≥ 3/≥4	96/78	93/99	87/99	98/90	NR	0.94/0.89	MIBC detection	3T
Piramide et al. [30]	133	VI–RADS (mp/bp)	≥ 3	86/76	95/93	75/67	97/95	93/90	0.90/0.85	MIBC detection	1.5T/3T
Ueno et al. [31]	91	VI–RADS	≥ 3/≥4	83/74	77/94	80/93	80/77	80/84	0.88	MIBC detection	1.5T/3T
van Koeverden et al. [32]	360	VI–RADS + biopsy	1–2 vs. 3–5	Ongoing	Ongoing	Ongoing	Ongoing	Ongoing	Ongoing	Treatment decision	3T
Al–Zubaidi et al. [33]	75	18 F–FDG PET/CT vs. CT	SUV	60/47	84/100	60/100	84/82	NR	NR	Nodal mets	NR
PET/CT Studies											
Bertolaso et al. [34]	130	18 F–FDG PET/CT vs. CT	RECIST/SUV	81/27	54/83	NR	NR	NR	NR	Nodal mets	NR
Einerhand et al. [35]	237 vs. 286	FDG PET/CT vs. CE–CT	RECIST/SUV	23/15	92/93	42/33	83/81	79	77	Nodal mets	NR
Longoni et al. [36]	199	18 F–FDG PET/CT	SUV	30	91	57	77	74	NR	Nodal mets	NR
Moussa et al. [37]	300	18 F–FDG PET vs. CT	SUV/size	40/13	80/86	61/45	62/55	NR	NR	Nodal mets	NR
Ng et al. [38]	16	18 F–fluciclovine PET	Visual	13	91	40	NR	67	NR	Nodal mets	NR
Pihl et al. [39]	157	18 F–FDG PET/CT	Visual	50	84	61	76	72	NR	Nodal mets	NR
Unterrainer et al. [40]	51	68Ga/18F–FAPI PET	SUV	82/100	90/82	65/47	96/100	NR	0.85/0.92	Nodal mets	NR
van Ginkel et al. [41]	92	18 F–FDG PET/CT	SUV	36	93	64	79	77	0.64	Nodal mets	NR
Gresser et al. [42]	391	Radiomics (LN seg)	Radiomics sig	73	84	64	91	NR	0.81	Nodal mets	NR

Abbreviations: *Acc* accuracy, *ADC* apparent diffusion coefficient, *APTw* amide proton transfer weighted, *AUC* area under the curve, *bp* biparametric, *CE–CT* contrast–enhanced computed tomography, *FAPI* fibroblast activation protein inhibitor, *FDG* fluorodeoxyglucose, *LN* lymph node, *MI* muscle invasion, *MIBC* muscle–invasive bladder cancer, *mp* multiparametric, *NR* not reported, *NPV* negative predictive value, *PPV* positive predictive value, *PUC* primary urothelial carcinoma, *Sens* sensitivity, *Spec* specificity, *SUV* standardized uptake value, *VUC* variant urothelial carcinoma, *VI–RADS* Vesical Imaging–Reporting and Data System.

The largest validation cohorts reinforced these findings across differing patient populations and institutions across North Africa, Asia and Europe, showing consistent findings. The three largest cohorts, Metwally et al. (*n*= 331) [28], Özdemir et al. (*n*= 283) [30], and Gong et al. (*n*= 198) [18], all demonstrated high sensitivity and specificity, with reported area under the curve (AUC) values up to 0.94, demonstrating robust discriminative performance across heterogeneous clinical environments.

Studies used either a 1.5 Tesla magnet, 3.0 Tesla magnet, or a mix between the two. When evaluating the median AUC for the two differing magnets, we found it was 0.89 versus 0.91, respectively.

### Biparametric Versus Multiparametric MRI

Three studies directly compared biparametric (bp–MRI) and multiparametric MRI (mp–MRI), revealing nuanced performance across differing patient populations [14, 29, 33]. In variant urothelial carcinoma, Arita et al. (2024)demonstrated mp–MRI superiority with sensitivities of 77–80% versus 65–67% for bp–MRI, yielding AUC differences of 0.03–0.04 in favor of the multiparametric approach [11].

Conversely, when evaluating all types of urothelial carcinoma regardless of varying histology, Bricio et al. (2022) found essentially equivalent performance between protocols, with overlapping AUC ranges (mp–MRI: 0.89–0.92; bp–MRI: 0.88–0.92) [14]. Similarly, Piramide et al. (2024) reported modest differences favoring mp–MRI (AUC 0.90 vs. 0.85) but with substantially overlapping confidence intervals [29].

### Enhanced VI–RADS Approaches

Three distinct augmentation strategies—morphologic parameters, tumor shape characteristics, and amide proton transfer weighted (APTw) enhancement—demonstrated significant improvements over the standard VI–RADS protocols, representing promising directions for enhanced bladder cancer staging accuracy.

Gong et al. (2024) evaluated additional morphological measurements including tumor contact margin, tumor longitudinal length, and tumor cellularity index in 198 patients [18]. VI–RADS combined with morphometric parameters achieved superior diagnostic performance with AUCs of 0.91 compared to 0.80–0.86 for VI–RADS alone.

Regarding tumor shape characteristics, Dogan et al. (2025) investigated sphericity, surface area, and elongation in 119 patients using 3D tumor segmentation [16]. Tumor sphericity, a measure of how closely a tumor approximates a sphere versus an irregular or infiltrative shape, was related to muscle invasion; however, it did not significantly increase the predictive performance of the algorithm.

Kong et al. (2024) incorporated APTw imaging alongside apparent diffusion coefficient (ADC) parameters in 106 patients [26]. When evaluated individually, both APTw values (*p* = 0.003) and ADC measurements (*p* = 0.020) enhanced the discriminative ability of the standard VI–RADS scores, achieving AUCs of 0.84–0.93 when combined, compared with the baseline VI–RADS AUC.

### Ongoing VI–RADS Clinical Trial

The BladParadigm study (van Koeverden et al., 2025) represents a pivotal randomized controlled trial evaluating mp–MRI–guided diagnostic pathways in clinical practice [32]. This Dutch multicenter non–inferiority study (*n* = 360) randomizes patients with suspected MIBC to either standard TURBT or a VI–RADS–guided pathway incorporating same–day tissue confirmation biopsy, with a primary endpoint of 2–year progression–free survival.

### PET–Based Molecular Imaging

Lymph node metastases are identified histologically in approximately 20% to 25% of patients at the time of radical cystectomy with pelvic lymph node dissection, and their presence is among the strongest predictors of disease recurrence and cancer–specific mortality, making accurate preoperative nodal assessment critical for treatment planning [4].

Nine studies encompassing 1,257 patients evaluated PET–based approaches for lymph node metastasis detection, representing an important complementary modality to anatomical imaging [34–42]. Performance demonstrated the characteristic pattern of molecular imaging with PET/CT achieving median sensitivity of 40% (range 13–100%) and specificity of 90% (range 54–93%).

Four studies directly compared 18 F–FDG PET/CT with contrast–enhanced CT for nodal staging [34–36, 38]. Across these comparative investigations, PET consistently demonstrated superior sensitivity for detecting nodal metastases (range 23–81%) compared to CT alone (range 13–47%), while maintaining comparable specificity. Notably, the false positive rate of FDG PET/CT ranged from 8% to 46% across studies, reflecting the known limitation of FDG uptake in inflammatory and reactive lymph nodes.

Two pilot investigations explored novel PET tracers. Ng et al. (2025) evaluated 18 F–fluciclovine PET/CT in 16 patients, achieving 13% sensitivity but 91% specificity for nodal detection [39]. Unterrainer et al. (2025) investigated 68Ga–FAPI PET/CT and 18 F–FAPI PET/CT, demonstrating substantially higher sensitivity (82% and 100%, respectively) with specificities of 90% and 82%, representing the most promising results for nodal staging in this review [41].

### Machine Learning and Radiomics

One study evaluated CT–based radiomics for lymph node metastasis detection. Gresser et al. (2023) analyzed 391 patients using machine learning algorithms applied to CT imaging features [42]. The combined radiologist plus radiomics model achieved superior diagnostic performance (AUC 0.81) with 73% sensitivity and 84% specificity compared to radiologist assessment alone.

### Quality Assessment

Risk of bias was assessed independently using the Quality Assessment of Diagnostic Accuracy Studies–2 (QUADAS–2) tool (Table 3). Overall methodological quality was high. Across all 33 studies, 23 (69.7%) were rated as low risk of bias across all four domains. No study was rated as high risk of bias in any domain. The most common source of potential bias was the index test domain, where nine studies (27.3%) received unclear ratings, primarily among PET/CT studies where reader blinding to pathology results was not clearly specified and one VI–RADS study with unclear blinding. In the flow and timing domain, one study (3.0%) received an unclear rating due to insufficient reporting of the interval between imaging and reference standard. Applicability concerns were low across all studies, with one study (3.0%) receiving an unclear rating for the reference standard domain due to use of a composite reference standard.

**Table 3:** QUADAS–2 risk of bias and applicability concerns summary

Study	Risk of Bias				Applicability Concerns		
	Patient Selection	Index Test	Reference Standard	Flow and Timing	Patient Selection	Index Test	Reference Standard
VI–RADS Studies							
Ahn et al. [10]	Low	Low	Low	Low	Low	Low	Low
Arita et al. [11]	Low	Low	Low	Low	Low	Low	Low
Arita et al. [12]	Low	Low	Low	Low	Low	Low	Low
Aslan et al. [13]	Low	Low	Low	Low	Low	Low	Low
Bricio et al. [14]	Low	Low	Low	Low	Low	Low	Low
Del Giudice et al. [15]	Low	Low	Low	Low	Low	Low	Low
Dogan et al. [16]	Low	Low	Low	Low	Low	Low	Low
Elshewy et al. [17]	Low	Low	Low	Unclear	Low	Low	Low
Gong et al. [18]	Low	Low	Low	Low	Low	Low	Low
Gupta et al. [19]	Low	Low	Low	Low	Low	Low	Low
Gungor et al. [20]	Low	Unclear	Low	Low	Low	Low	Low
Huele et al. [21]	Low	Low	Low	Low	Low	Low	Low
Islam et al. [22]	Low	Low	Low	Low	Low	Low	Low
Jai–ua et al. [23]	Low	Low	Low	Low	Low	Low	Low
Kazan et al. [24]	Low	Low	Low	Low	Low	Low	Low
Kim et al. [25]	Low	Low	Low	Low	Low	Low	Low
Kong et al. [26]	Low	Low	Low	Low	Low	Low	Low
Liu et al. [27]	Low	Low	Low	Low	Low	Low	Low
Metwally et al. [28]	Low	Low	Low	Low	Low	Low	Low
Özdemir et al. [29]	Low	Low	Low	Low	Low	Low	Low
Piramide et al. [30]	Low	Low	Low	Low	Low	Low	Low
Ueno et al. [31]	Low	Low	Low	Low	Low	Low	Low
van Koeverden et al. [32]	Low	Low	Low	Low	Low	Low	Low
Al–Zubaidi et al. [33]	Low	Unclear	Low	Low	Low	Low	Low
PET/CT Studies							
Bertolaso et al. [34]	Low	Unclear	Low	Low	Low	Low	Low
Einerhand et al. [35]	Low	Unclear	Low	Low	Low	Low	Low
Longoni et al. [36]	Low	Unclear	Low	Low	Low	Low	Low
Moussa et al. [37]	Low	Unclear	Low	Low	Low	Low	Low
Ng et al. [38]	Low	Low	Low	Low	Low	Low	Low
Pihl et al. [39]	Low	Unclear	Low	Low	Low	Low	Low
Unterrainer et al.[40]	Low	Unclear	Low	Low	Low	Low	Low
van Ginkel et al. [41]	Low	Unclear	Unclear	Low	Low	Low	Unclear
Gresser et al. [42]	Low	Low	Low	Low	Low	Low	Low

Abbreviations: *QUADAS–2* Quality Assessment of Diagnostic Accuracy Studies–2. Risk of bias and applicability concerns rated as Low, High, or Unclear for each domain.

Among the VI–RADS studies, methodological quality was particularly strong: 21 of 23 studies (91.3%) were rated as low risk of bias across all domains, with the majority using prospective or retrospective cohort designs, blinded readers, pre–specified VI–RADS thresholds, and appropriate reference standards (TURBT or radical cystectomy pathology). PET/CT studies demonstrated lower certainty in the index test domain due to inconsistent reporting of reader blinding.

## Discussion

The substantial variation in VI–RADS sensitivity (65–100%) and specificity (23–100%) across studies likely reflects fundamental differences in radiologist expertise rather than inherent limitations of the scoring system. Studies with multiple readers with differing levels of experience demonstrated that radiologist training and experience significantly influenced diagnostic accuracy, suggesting that standardized training protocols could narrow this performance gap.

Multiparametric protocols demonstrated superior sensitivity while maintaining comparable specificity for variant urothelial carcinoma; in this population mp–MRI is optimally suited. However, bp–MRI has been shown in some cases to be comparable in diagnostic accuracy, which offers resource–conscious alternatives for facilities lacking dynamic contrast–enhanced capabilities.

The dilemma of VI–RADS is that using a cutoff score of three for MIBC has translational challenges. Using the cutoff score of three for MIBC risks potentially premature cystectomy or inadequate staging, while using the cutoff score of four increases specificity at the cost of potentially missing cases. The uncertainty at the threshold boundary, particularly for VI–RADS score 3 lesions, represents the most critical area for clinical decision–making and argues for institutional protocols that integrate VI–RADS with clinical factors and potentially incorporate molecular subtyping and other tissue–based biomarkers.

CT remains the standard imaging modality for lymph node assessment in MIBC, but its characteristically poor sensitivity (13–47%) creates a clinically significant problem of delayed time to treatment. When CT misses smaller metastatic deposits due to size–based criteria, patients may undergo inappropriate treatment sequencing.

18 F–FDG addresses this limitation through metabolic assessment, achieving improved sensitivity (23–81%) by detecting metabolically active disease regardless of lymph node size. While sensitivity remains variable, the improvement represents a meaningful clinical advantage.

The most promising development emerged from fibroblast activation protein inhibitor studies, with FAPI tracers achieving substantially higher sensitivity (82–100%) while maintaining excellent specificity (82–90%). This suggests that further investigation should be done on these novel tracers and on larger scales.

For institutions unable to implement PET scanning due to resource constraints, radiomics machine learning approaches applied to standard CT demonstrated competitive performance (73% sensitivity, 84% specificity) when combining automated lymph node segmentation with radiologist interpretation, offering a scalable alternative for nodal staging.

The substantial cost and infrastructure requirements of advanced imaging technology create significant deployment barriers, particularly in resource–limited settings worldwide. However, the geographic diversity of included studies spanning East Asia, India, Europe, Egypt, Lebanon, and Turkey demonstrates that VI–RADS implementation is feasible across varying healthcare systems.

The most clinically viable approaches integrate seamlessly into existing imaging workflows without substantial additional costs or infrastructure investment. Morphometric parameter assessment builds upon the standard mp–MRI protocols already in use, requiring only additional measurement time rather than new equipment or sequences.

While ideal implementation would involve PET scanning with novel tracers and mp–MRI with APTw diffusion at every center, clinical reality demands more pragmatic solutions, which can be attained with bp–MRI with morphological feature grading and machine learning analysis of standard CT, representing the most implementable advances for widespread adoption.

This study has several limitations. The majority of included studies were retrospective in design, introducing potential selection bias. Heterogeneity in imaging protocols, reader experience, and reference standards across studies limits direct comparison of diagnostic performance metrics. The VI–RADS literature is predominantly composed of single–institution studies with variable sample sizes, and the PET literature remains limited to small cohorts for the most promising novel tracers. To our knowledge, only one randomized controlled trial is currently underway. Additionally, publication bias may favor studies with positive results. Future prospective, multicenter studies with standardized protocols are needed to validate these findings.

## Conclusions

Novel imaging modalities demonstrate significant potential for improving staging accuracy prior to surgical management of invasive bladder cancer. VI–RADS provides a reproducible, standardized framework for MRI–based muscle invasion assessment, with threshold selection allowing clinicians to balance sensitivity and specificity according to clinical context. PET–based molecular imaging, particularly with emerging FAPI tracers, substantially improves nodal staging sensitivity beyond the limitations of conventional CT size–based criteria. CT–based radiomics offers a scalable alternative for institutions without advanced imaging infrastructure. Integration of these complementary approaches into preoperative staging pathways may optimize patient management, including use or omission of neoadjuvant therapy and consideration for organ–sparing strategies, and improve surgical planning. Prospective multicenter validation studies, including the ongoing BladParadigm trial, are needed to confirm these findings and establish evidence–based imaging protocols for clinical practice.

## Key References

- Arita Y, Kwee TC, Woo S, et al. Biparametric versus multiparametric magnetic resonance imaging for assessing muscle invasion in bladder urothelial carcinoma with variant histology using the Vesical Imaging-Reporting and Data System. *Eur Urol Focus.* 2024;10(1):131-138.
  - ◌ Demonstrates that biparametric MRI may perform comparably to multiparametric MRI for evaluating muscle invasion in bladder cancers with variant histology, suggesting a potential reduction in examination complexity and contrast use without compromising diagnostic performance.
- Piramide F, Sica M, Fondello G, et al. Use of multiparametric and biparametric magnetic resonance imaging in bladder cancer staging: prospective observational study and analysis of radiologist learning curve. *J Clin Med.* 2024;13(22):6955.
  - ◌ Provides prospective evidence that both mpMRI and bpMRI can accurately stage bladder cancer while also demonstrating the reproducibility of VI-RADS interpretation across different levels of reader experience, supporting broader clinical implementation.
- Al-Zubaidi M, Ong K, Viswambaram P, et al. Comparing fluorodeoxyglucose positron emission tomography with computed tomography in staging for nodal and distant metastasis in urothelial/bladder cancer. *BJUI Compass.* 2024;5(4):473-479.
  - ◌ Highlights the incremental value of FDG-PET/CT over conventional CT for detecting nodal and distant metastatic disease, supporting its increasing role in preoperative staging of muscle-invasive bladder cancer.
- Unterrainer LM, Schmid HP, Kunte SC, et al. ^68Ga-FAPI and ^18F-FAPI PET/CT for detection of nodal metastases prior radical cystectomy in high-risk urothelial carcinoma patients. *Eur J Nucl Med Mol Imaging.* 2025. doi:10.1007/s00259-025-07239-6.
  - ◌ One of the first clinical studies evaluating FAPI PET/CT in bladder cancer, demonstrating the potential of a novel molecular imaging tracer to improve nodal staging beyond FDG-based imaging.
  - ◌ Represents an emerging direction in molecular imaging that may influence future staging algorithms for high-risk urothelial carcinoma if validated in larger prospective studies.

## Data Availability

No datasets were generated or analysed during the current study.

## Declarations

This article does not contain any studies with human or animal subjects performed by any of the authors.
